# Preterm birth and metabolic implications on later life: A narrative review focused on body composition

**DOI:** 10.3389/fnut.2022.978271

**Published:** 2022-09-15

**Authors:** Amanda Casirati, Alberto Somaschini, Michela Perrone, Giulia Vandoni, Federica Sebastiani, Elisabetta Montagna, Marco Somaschini, Riccardo Caccialanza

**Affiliations:** ^1^Clinical Nutrition and Dietetics Unit, Fondazione IRCCS Policlinico San Matteo, Pavia, Italy; ^2^Division of Cardiology and Cardiac Intensive Care Unit, San Paolo Hospital, Savona, Italy; ^3^Neonatal Intensive Care Unit, Fondazione IRCCS Ca’ Granda Ospedale Maggiore Policlinico, Milan, Italy; ^4^Clinical Nutrition, Fondazione IRCCS Istituto Nazionale Dei Tumori, Milan, Italy; ^5^Endocrinology and Metabolic Diseases, Azienda USL IRCCS di Reggio Emilia, Reggio Emilia, Italy; ^6^Unit of Neonatology, Sant’Anna Clinic, Lugano, Switzerland

**Keywords:** body composition, preterm, preschool, fetal programming, early nutrition, microbiota

## Abstract

Preterm newborn infants are characterized by low body weight and lower fat mass at birth compared with full-term newborn neonates. Conversely, at term corrected age, body fat mass is more represented in preterm newborn infants, causing a predisposition to developing metabolic syndrome and cardiovascular diseases in later life with a different risk profile in men as compared with women. Postnatal growth is a complex change in anthropometric parameters and body composition. Both quantity and quality of growth are regulated by several factors such as fetal programming, early nutrition, and gut microbiota. Weight gain alone is not an optimal indicator of nutritional status as it does not accurately describe weight quality. The analysis of body composition represents a potentially useful tool to predict later metabolic and cardiovascular risk as it detects the quality of growth by differentiating between fat and lean mass. Longitudinal follow-up of preterm newborn infants could take advantage of body composition analysis in order to identify high-risk patients who apply early preventive strategies. This narrative review aimed to examine the state-of-the-art body composition among born preterm children, with a focus on those in the pre-school age group.

## Introduction

Preterm birth (PB) may seriously affect postnatal growth and neurodevelopment and increase the risk of adverse outcomes from infancy to adulthood. Yearly, 15 million babies in the world are born before 37 weeks of gestation; in Europe, the estimated rate of PB ranges from 5 to 10% (1).

Although at birth preterm infants are characterized by a lower weight and fat mass (FM), at term corrected age, their body fat percentage is more represented if compared with full-term infants. This peculiar pattern of growth that occurs during the first weeks of life is a consequence of complex metabolic and endocrine adaptations at extrauterine life and is also affected by early nutrition. The enhanced importance of early life nutrition and optimization of growth in terms of body composition is mainly related to the advances in neonatal intensive care, which allow the survival of infants of lower gestational age. Indeed, rapid catch-up growth during infancy and childhood is related to an increased risk of developing non-communicable diseases, including cardiovascular diseases and metabolic syndrome in later life (2).

The study of body composition (BC) and growth patterns during early life allows us to better understand the longitudinal associations among body compartments, metabolic–endocrine pathways, and health consequences in the long term (3).

This review aimed to investigate the state-of-the-art scientific literature regarding BC analysis among preterm born children, focusing on pre-school age. To better understand how BC can predict metabolic risk in later phases of life, we examined the role of key factors related to BC, such as fetal programming, early nutrition, and gut microbiota.

Due to the wide heterogeneity across studies in terms of populations (by age, ethnics, and gender), terminologies (PB classifications based on birth weight vs. gestational age), and methodologies in measuring BC (2 or more compartments model-based), we provide a narrative review to avoid inappropriate comparisons of heterogeneous data.

## Challenges of preterm birth

### Prematurity: Concepts and definitions

Prematurity refers to a birth that occurs before 37 weeks of pregnancy (1). According to the gestational age (GA), PB is stratified into extremely preterm (GA < 28 weeks), very preterm (GA 28–32 weeks), moderate preterm (GA 32–34 weeks), and late preterm (GA 34–36 weeks), with increasing morbidity and mortality risk in lower GA (1).

PB is characterized by Low Birth Weight (LBW, birth weight < 2,500 g). Particularly, preterm infants could be categorized according to BW as Very Low Birth Weight (VLBW, BW < 1,500 g), Extremely Low Birth Weight (ELBW, BW < 1,000 g), and Micro Preemie (BW < 750 g). There are few concepts and definitions that may overlap and should be clarified when approaching PB. Small for Gestational Age (SGA) refers to the birthweight below the 10th percentile of recommended sex-specific birth weight for the referenced GA (1).

Intrauterine growth restriction (IUGR) is an abnormal fetal growth pattern referring to an impoverished fetal growth due to fetal, maternal, or placental factors (4). IUGR reflects fetal distress, whereas SGA only provides a punctual measure of weight and not a longitudinal measure of prenatal growth. Extrauterine Growth Restriction (EUGR) is historically diagnosed when weight is < 10th percentile at either discharge or 36–40 weeks postmenstrual age. A paper published by Fenton pointed out how the punctual definition of EUGR is useless if not harmful. Indeed, considering that this kind of definition is based on an arbitrary statistical growth percentile cutoff and is based only on weight at 36–40 weeks post-conceptional age without any consideration of birth weight percentile, there is an increased risk of overdiagnosis of growth deviation. This could lead to an incorrect understanding of growth patterns, which in turn, may lead to overfed practice, increasing the risk for non-communicable diseases later in life (5).

### The fetal programming

Several studies suggest that metabolic and cardiovascular risk originates during intrauterine life and it is related to the programming of cardiovascular and metabolic functions (6). The programming concept refers to the hypothesis that endogenous or environmental stimuli occurring during pregnancy may permanently affect both the fetus and the infant lifelong (7).

**FIGURE 1:**
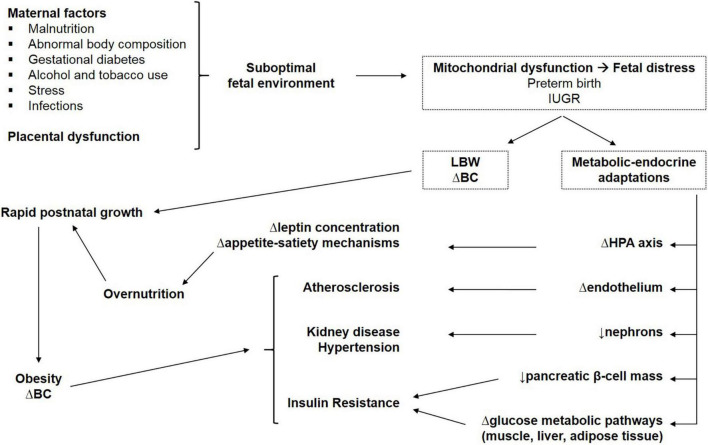
Potential mechanisms explaining the relationship between LBW and metabolic consequences in adult life. IUGR, intrauterine growth restriction; LBW, low birth weight; BC, body composition; Δ, altered; HPA, hypothalamic–pituitary–adrenal.

Barker hypothesized that small size at birth has a causal relationship with the development of hypertension, coronary heart disease, and non-insulin-dependent diabetes, in middle age. LBW infants considered in his study were mainly SGA and IUGR because the mortality rate of premature newborn infants was very high at that time (8) [Barker1990]. The fetus is dependent on the transfer of nutrients from the mother and adapts to an inadequate nutrient supply in a number of ways: prioritization of brain growth at the expense of other tissues such as the abdominal viscera, reduced secretion of and sensitivity to insulin, low nephron number, upregulation of hypothalamic–pituitary–adrenal axis, low muscle mass, and altered arterial structure (9). Barker’s “fetal programming” (FP) or Developmental Origin of Health and Disease (DOHaD) hypotheses proposed that although occurring in response to a transient phenomenon (fetal undernutrition) these changes become permanent or “programmed” because they occur during a critical period of development. The potential mechanisms explaining the relationship between LBW and diseases in adult age are summarized in Figure 1.

Initially attributed solely to SGA and IUGR infants, the phenomenon of FP has subsequently been expanded to include preterm infants (10). The maternal–placental unit supports fetal growth through a constant flow of nutrients and hormones; PB disrupts this metabolic–endocrine unit when organs maturation is not fully achieved, with an interruption of the normal organogenesis of multiple organ systems including the lungs, pancreas, kidney, liver, and vascular tree. In addition, various antenatal (maternal infections, hypertension, preeclampsia, gestational diabetes, and use of medications), perinatal, and postnatal factors (hypoxia, hyperoxia, sepsis, hypotension, thrombosis, bleeding, and acute kidney injury) have been associated with impaired organ development in the preterm neonate.

The consequences of PB on angiogenesis and kidney development are among the most studied. PB leads to an interruption in angiogenesis, which causes altered patterns of development of the vascular tree that is apparent as early as infancy. Data from long-term follow-up studies have shown an inverse relationship between gestational age and risk of adult hypertension in both men and women who were born preterm (11). Alterations of vascular function and structure related to PB and increased risk of late cardiovascular disease include impaired endothelial function, a reduction in microvascular density, and reduced arterial dimensions and arterial elasticity (12, 13). Specifically, endothelial dysfunction, particularly impaired endothelial-dependent arterial relaxation, is considered an early marker for the development of hypertension in LBW infants born preterm (14).

Regarding the impact of PB on kidney development, an important aspect is nephron endowment which is the number of nephrons that an individual has at birth. In the human fetus, nephrogenesis reaches completion at 34–36 weeks of gestation with more than 60% of nephrons being formed in the last trimester; therefore, nephron endowment is low in infants born preterm. In 1997, Brenner proposed the hyperfiltration theory, which hypothesized that early loss of nephrons mass results in hyperfiltration of remaining nephrons, proteinuria, progressive kidney injury, and increased risk of hypertension in later life (15). Preterm and critically ill neonates are highly susceptible to renal injury because of renal functional immaturity and incomplete nephrogenesis, and peripartum asphyxia, nephrotoxic drug exposure, and episodes of acute kidney injury can further impact nephron development.

### The role of epigenetic factors

Multifactorial diseases such as diabetes or hypertension are the result of complex interactions between genetic constitution and environmental factors that act from the fetal age to determine the adult phenotype. In the attempt to clarify the mechanisms of fetal programming, there is an emerging interest in the role of epigenetic modifications. Epigenetic changes can permanently modify gene expression without altering DNA sequence, are established in early development, and, once set, are mitotically heritable (16). Epigenetic changes include methylation of cytosine and modifications (acetylation and phosphorylation) of the histones, and they affect the transcriptional activity of genes. Unfavorable environmental factors may cause methylation and alter tissue function; several studies demonstrate that diet can have an impact on DNA methylation, in particular, a maternal protein-deficient diet led to global hypermethylation of DNA aimed to modulate energy metabolism (17).

### The impact of oxidative stress in preterm newborn neonates

Hospitalized preterm newborn infants require intensive care management (such as mechanical ventilation, oxygen administration, and blood transfusions) and frequently experience elevated oxidative stress and inflammation. Oxidative stress occurs when there is an imbalance between reactive oxygen species (ROS) and antioxidant systems and may contribute to significant pathology in the maternal–infant dyad. The downstream inflammation and cellular damage that results from oxidative stress have implications on the developing kidneys, lungs, and cardiovascular system. In immature newborns, exposure to supplemental oxygen may halt microvessel growth in the lung and retina, leading to serious short−term complications such as bronchopulmonary dysplasia and retinopathy of prematurity. In the developing kidney, the negative impact of oxidative stress related to fluctuating oxygen exposure from hyperoxia to intermittent hypoxia has also been implicated in the development of kidney injury in postnatal animal models (18). There is evidence that neonatal hyperoxia exposure increases ROS production and alters elastin and collagen distribution within the systemic vasculature with impaired endothelial function and reduced arterial dimensions and elasticity (19, 20).

### The catch-up growth

The aim of nutritional care in preterm infants is to promote a postnatal gain of weight which should follow the growth trajectory they would have in the womb, aiming to avoid growth failure and neurodevelopment delay (21, 22). Of note, both overnutrition and undernutrition at critical stages of development may have non-desirable consequences in preterm and infant with IUGR: while lowering weight gain velocity may delay mental performance, growing fast increases the risk of obesity, dyslipidemia, hypertension, insulin resistance, and type II diabetes in later life (6, 23). Balancing the short- and long-term effects of early feeding in this high-risk population has become a clinical dilemma for neonatologists, as short-term benefits may be outweighed by long-term risks (6, 24). Catch-up growth refers to a rapid compensatory gain of anthropometry (i.e., upward crossing weight centiles) and BC parameters occurring during infancy in LBW infants (25). Evidence is mounting to show that rapid catch-up growth, characterized by an increase in fat storage or an increase in BMI, leads to the development of higher blood pressure, insulin resistance, and cardiovascular risk already in childhood (26). According to the “thrifty phenotype” hypothesis, this rapid and marked fat accumulation improves thermoregulation, increases body energy stores allowing a better adaptation to the extrauterine life, and is regulated both by several endocrine factors (i.e., IGF-1, leptin, adiponectin) and early life nutrition (27, 28). Interestingly, the timing of catch-up growth appears to modulate the subsequent metabolic risk, as early catch-up seems more beneficial while later catch-up appears to be more harmful (29). In addition, the effects of catch-up growth may be different if the catch-up occurs predominantly in height (linear growth) or in weight, and, in most studies, the adverse effects were more marked among those who had been LBW or preterm and subsequently become overweight or obese (30). In this context, the analysis of BC provides a better understanding of nutritional state and is potentially more helpful in predicting adverse health outcomes in later life than birth weight alone, especially in preterm and SGA infants. Specifically, BC may investigate the quality of growth differentiating between lean and adipose tissue gain (31, 32).

## The added value of body composition

### Fundamentals

LBW has been often used as an index of inadequate fetal growth to predict the subsequent risk of comorbidities. Not only does BW influence the risk of non-communicable disease but as pointed out by Lucas et al. it plays an important role in the changes in size between birth and adulthood, which have a deeper impact on disease development than birth weight alone (7).

Anthropometry consists of height, weight, head circumference, body circumferences, and skinfold thickness. It is considered an applicable, inexpensive, and non-invasive method to study measurements and proportions of the human body (1). Anthropometric indices that mathematically combine body weight and length, for example, body mass index (BMI), are often used to assess nutritional status as they are practical, reproducible, and easy to calculate (33).

Weight, which is the sum of several different compartments (skeletal muscle, lean mass, fat mass, water content, and bone), is the key parameter for assessing growth velocity. The two methods most frequently used are grams per kilogram per day according to Patel’s exponential model (EM), which uses weights from 2-time points (34) and Fenton growth charts based on the change in weight z-score (35). Patel’s EM was used to assess the growth of preterm newborn infants during hospitalization (36).

However, when body weight modifies, a standard scale does not detect which tissue and how much changes. Therefore, the weight alone does not accurately reflect the neonatal quality of growth. Moreover, considering the high hydration rate of infants, weight is strongly affected by fluid status (37). In addition, anthropometry does evaluate fat distribution: for instance, it is well known that central abdominal fat is most harmful than subcutaneous fat (38, 39).

**FIGURE 2:**
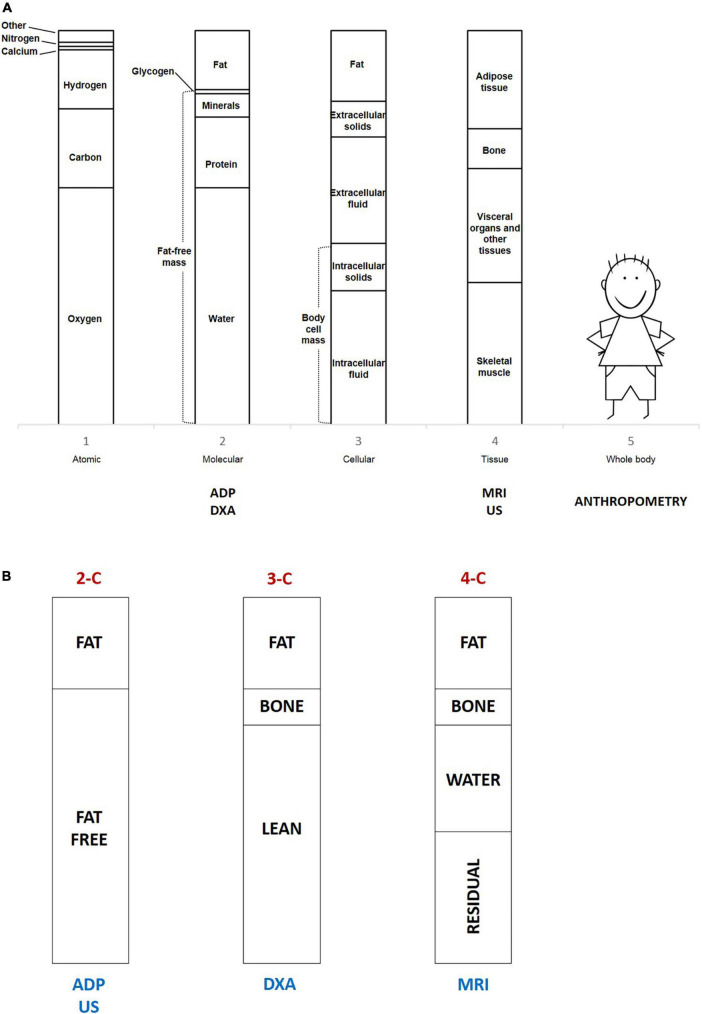
**(A)** The five levels describe body composition and the different techniques used according to levels. ADP, air displacement plethysmography; DXA, dual-energy x-ray absorptiometry; MRI, magnetic resonance imaging; US, ultrasound imaging. **(B)** The multi-compartment models and the different techniques used according to models. 2-C, two-compartment model; 3-C, three-compartment model; 4-C, four-compartment model; ADP, air displacement plethysmography; DXA, dual-energy x-ray absorptiometry; MRI, magnetic resonance imaging; US, ultrasound imaging.

BC is the best long-term indicator of nutritional status both in the state of health and disease (40). Wang et al. defined BC by a model of five levels (atomic, molecular, cellular, tissue, and whole body), as graphically shown in Figure 2A. The sum of water, proteins, minerals, and glycogen defines the so-called fat-free mass (FFM), considering the molecular level (level 2). The two-compartment model (2-C) basically differentiates body weight between FM and FFM, considering body weight as the sum of FM and FFM (41, 42). This model was originally developed to assess body fatness, defined as body fat mass percentage (FM%) on the total body weight, but ignores the heterogeneity of the compartments. Advanced multiple-compartment models divide FFM into various components (43, 44). The three-compartment model (3-C) describes body weight as the sum of FM, bone mineral content, and lean components (skeletal muscle and residual). According to the four-compartment (4-C) model, BW is the sum of FM, water (intracellular and extracellular), bone mineral content, and residual mass. Figure 2B summarizes the multi-compartment models. The cellular level (level 3) identifies body cell mass (BCM), which is the metabolic active fat-free portion of the intracellular space. The tissue level (level 4) differentiates between adipose tissue and non-adipose tissues (skeletal muscle, bone, and residual tissues) (41). FFM is often improperly termed as lean body mass (LBM), nevertheless, lean mass is only a part of FFM. In the same way, lean soft tissue is a part of non-adipose tissue (42). Body weight loss and BMI reduction only inconstantly reflect FFM loss. To assess changes in BC associated with weight variations, the 4-C model is considered the most accurate method (42, 45). The analysis of BC represents a useful marker of later metabolic risk as it distinguishes FM from other components of body weight (41).

### Techniques to assess body composition in preterm newborn infants

BC assessment in preterm infants is challenging as growth patterns are not linear. Furthermore, PB can alter the pattern of adiposity, with an increased risk of developing metabolic diseases (38). For this reason, quality is as important as the quantity of growth (3).

Studies measuring preterm body composition are mostly performed when infants reach term equivalent age (TEA), referred to as the 40th week of gestation. At TEA, preterm infants have been shown to have decreased amounts of FFM and increased FM% when compared with term infants of the same GA (39).

Several methods allow us to measure the different tissues or compartments. Currently, most studies use air displacement plethysmography (ADP) and dual-energy x-ray absorptiometry (DXA), while only a few studies have applied MRI and ultrasound imaging (US).

Unfortunately, due to the wide heterogeneity across methodologies and assumptions, body composition values collected using different methods cannot be compared (3). Moreover, there are neither uniform reference data for BC of preterm infants nor a consensus on the preferred method to use (33). However, ADP revealed to be highly suitable for monitoring changes of BC in preterm neonates in both research and clinical settings (46, 47), and it has been used to generate BC reference curves in term infants (44).

#### Air-displacement plethysmography

ADP is based on the principle of hydrostatic weighing using air instead of water. In an environmentally controlled chamber, the system rapidly provides a measure of infant BC with a two-compartment model, as it calculates FFM and FM from body mass measurements and body volume. ADP was revealed to be a rapid, non-invasive and reliable technique to measure BC in infants both in research and clinical setting (43). Roggero and colleagues firstly compared FM% values in preterm infants obtained with ADP with the reference H_2_ (18) O dilution technique and founded ADP precise and accurate (mean difference 0.32 ± 1.57, *p* = 0.53) (46). Measurements by ADP are independent of the body size and fatness (43) and are not affected by behavioral states of infant (such as quiet and alert, awake and active, and crying intensely) and urination (48). Moreover, from a comparison of two different ADP devices, it has been reported an excellent inter-device reliability in measuring FM% (mean difference 0.15, *p* = 0.34) (46). However, fat distribution cannot be determined by this technique (33, 49, 50).

#### Dual-energy x-ray absorptiometry

DXA is based on the emission of low- and high-energy x-rays toward each point of the body pixel and then evaluates the x-ray attenuation as they pass through the tissues. It is a quick, reliable, and repeatable method and provides information about regional FM, non-bone lean mass, and bone mineral content and density, according to a three-compartment model. However, it has some critical issues. It exposes subjects to a small quantity of radiation, it may overestimate body fat, and it requires both expensive machines and specifically skilled investigators. ADP and DXA provide data on fat mass, which is different from adipose tissue (51, 52); in fact, fat is a molecular component referring to triglycerides, and it is mainly found in adipose tissue, but it is also present in other tissues. Adipose tissue includes 80% of fat, the remaining 20% is composed of water, protein, and minerals (51). Of note, DXA seems to underestimate trunk fat and overestimate limb lean mass because of the poor accuracy in differentiating between fat and fat-free tissue in these body regions (53). As in infants, the trunk is overrepresented while limbs are smaller in proportion, regional estimation of FM in this population could be imprecise.

#### Magnetic resonance imaging

MRI is a four-compartment technique. It estimates the volume of adipose tissue and gives information about lean body mass, water, and bone mass either per body area or in the whole body. It is considered a reliable method for the evaluation of subcutaneous and visceral fat. MRI technique quantifies adipose tissue volume as voxels or volume elements. MRI detects both adipose tissue depots and ectopic fat in the liver and skeletal muscle (6). It is a non-invasive method, and it provides a highly reproducible and accurate assessment of adipose tissue volume as it differentiates between intra-abdominal and subcutaneous adipose tissue. However, it is a time-consuming and expensive method. Moreover, infants must be sedated during the examination as movements reduce the quality of measurements, and they have to be transferred from the Neonatal Intensive Care Units to undergo the examination (33, 44, 54).

#### Ultrasound imaging

US is another emerging technique used to measure regional lean tissues and FM according to the two-compartment model (33). The US waves cross body tissues and are reflected depending on the density and texture of the different tissues (fat, muscle tissue, or bone) encountered by the beams. US imaging can measure regional lean mass and fat mass without any x-ray exposure or need for sedation. Muscle thickness and subcutaneous fat thickness are evaluated in two proximal (biceps brachii and quadriceps femoris) and one distal (anterior tibial) muscle groups (55). There is good repeatability, but the machine is expensive and the exam is time-consuming (33). Furthermore, US demands very experienced investigators in order to manage the pressure exerted by the transducer on the skin and to identify the correct anatomical sites (56).

## Impact of preterm birth and weight at birth on body composition

Organ growth and development begin soon after conception when the fetus is composed only of FFM. FM accumulation occurs later in pregnancy, with the aim of enhancing the chance of survival of newborns after the interruption of maternal supply at birth (57). After birth, an excessive FM gain during early infancy increases the risk of further development of non-communicable diseases, especially if the fat distribution is abnormal (58).

### Development of adipose tissue

From the embryonic stage, human tissues can synthesize lipids using glucose which contributes to increasing the fetal adipose tissue. During early pregnancy, insulin resistance and estrogen effects increase maternal fat depots and adipose tissue breakdown with subsequent hypertriglyceridemia. All fatty acids (FAs) can provide energy by mitochondrial fatty acid oxidation pathway, but polyunsaturated fatty acids of the n-3 or n-6 series are the preferential substrate essential for structural and metabolic intrauterine development. Most FAs from the maternal circulation are esterified and associated with lipoproteins, which are taken up by the placenta and hydrolyzed by lipases. Fetal fat accretion during the last weeks of gestation occurs very rapidly and is sustained by FAs crossing the placenta and fetal lipogenesis (59).

Development of adipose tissue begins during the second trimester of intrauterine life and it is a dynamic process, continuing lifelong. This process involves both mechanisms of hypertrophy (size enlargement of adipocyte cells) and hyperplasia (increases in the number of adipocytes within a lobule). At 14 weeks of gestation, the first fat depots can be detected, and the primitive fat lobules develop. The number of lobules increases until about 23 weeks GA. Successively, until 29 weeks GA, adipose tissue grows as fat lobule size increases, which continues throughout postnatal life, while the fat lobule number remains constant. During the last 10 weeks of gestation, fat mass increases linearly with GA, especially in subcutaneous adipose tissue (60). It has been estimated a lipid accretion rate of 7.8 g/day at 24–28 weeks GA vs. increases up to 19.8 g/day at 36–40 weeks GA (61).

Overall, in full-term infants, FM growth is linear with the increase in fat cell size during the first months of life, whereas the number of fat cells remains stable. Conversely, PB alters the ideal growth pattern and disrupts the ontogeny development of BC; in addition, preterm infants experience a difficult absorption of fatty acids due to the immaturity of the gastrointestinal tract (62).

BC assessment performed using ADP shows lower FM in preterm LBW infants as compared with full-term infants, due to the fact that PB prevents the opportunity to accumulate sufficient reserves of adipose tissue *in utero* (27, 31), leading to low energy and nutrient reserves (63). As a consequence, an adequate nutritional program is necessary to guarantee a sufficient development of FM and FFM in an immature infant (64).

### Altered body composition development in the first phases after birth in the preterm

In an American cohort of 218 late preterm neonates (mean GA at birth 33.8 ± 1.9 weeks, mean birth weight 2,190 ± 494 g), mean FM% was 8.3 ± 4.1 and FFM 1.88 ± 0.4 kg (39). Giannì et al. found that late preterm (mean birth weight 2,153 ± 269 g) had mean FM% of 4.7 ± 2.7 at birth (65).

TEA is an important landmark point to evaluate BC and may reveal the disparities of extrauterine development of preterm infants who are exposed to an unfavorable environment compared to the fetal development *in utero* of full-term newborns, predisposing to metabolic syndrome and cardiovascular disease in childhood and adulthood. The meta-analysis of Johnson et al. showed that, at TEA, preterm infants have greater FM measured with ADP by 6.3% (95% CI 5.36–7.32%, *p* < 0.001) compared with full-term infants (52).

In an Italian cohort, mean FM% was found to be significantly higher in preterm neonates (mean GA at birth 29.9 ± 2.3 weeks, mean birth weight 1,118 ± 274 g) at TEA, as compared with term neonates (14.8 ± 4.4 vs. 8.6 ± 3.7, *p* < 0.001) (66). In a Dutch cohort at TEA, preterm infants (mean GA at birth 29 ± 1.6 weeks, mean birth weight 1,170 ± 316 g) presented a mean body fat percentage of 20.7 (18.4–23.0) and a mean FFM of 3,309 ± 462 g. FM% of males and females [21.2 (18.4–23.4) and 20.4 (18.1–22.6)] was not significantly different (*p* = 0.594), differently from FFM values (3,447 ± 430 g and 3,114 ± 442 g, *p* = 0.006) (2).

It has been demonstrated that FM is negatively correlated with GA and positively associated with weight gain after birth (66). Considering BC analysis according to GA, Giannì et al. reported that late preterm infants (mean birth weight 2,423 ± 333 g), have more FM% (14.2 ± 4.5 vs. 8.9 ± 2.9, *p* < 0.001) at TEA in comparison with full-term newborns at birth (65). Similar observations were made by Chmielewska et al. who reported that very preterm infants (mean birth weight 1,136 ± 406 g) have a significantly greater FM% (20.2 ± 3.6 vs. 11.7 ± 3.9, *p* < 0.001) at TEA compared with full term (67). Bruckner et al. found that extremely preterm infants (mean birth weight 779 ± 149 g) have a significantly higher FM% compared to very preterm infants [17.0 (15.9–18.1) vs. 15.5 (14.7–16.2), *p* = 0.034] at TEA (68). All three authors assessed BC using air displacement plethysmography systems.

It has been reported a decrease in subcutaneous adipose tissue and an increase in visceral fat in preterm infants assessed by magnetic resonance imaging at TEA (69, 70), which persists later at further evaluations (69). In addition, they appear shorter in length and characterized by lower lean mass as compared to term born infants (65, 67, 71).

**FIGURE 3:**
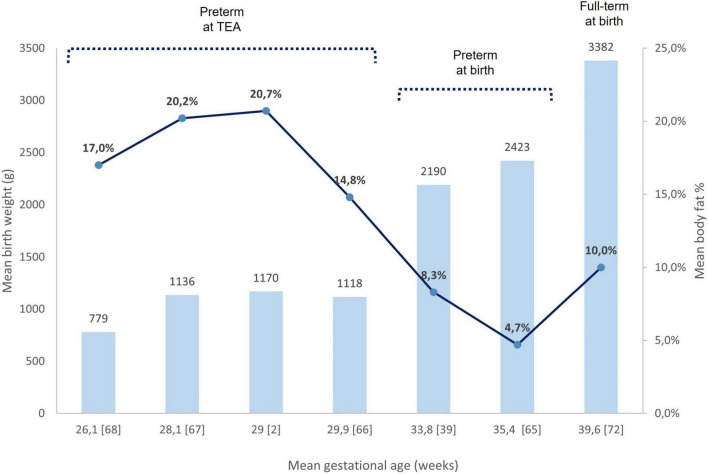
Birth weight and body fat% assessed by ADP. Comparison between preterm neonates at birth, preterm at TEA, and full-term at birth. Each column refers to a bibliography reference [number]. The mean birth weights of each study are reported upper their respective column. Mean body fat% in each study is represented as a blue circle on the respective column. ADP, air displacement plethysmography; TEA, term equivalent age.

In a meta-analysis including 6,231 full-term born infants, mean FM% and FFM were 10.0 ± 4.1 and 2,883 ± 356 g, respectively, at birth (72). The analysis of BC showed a great degree of variability for FM% according to socioeconomic background and ethnicity, ranging from 7.8% in Ethiopia (East-African race) to 13.6% in the USA (14.3% in Hispanic infants and 11.2% in African American infants). In addition, both maternal nutritional status and diet during pregnancy may have an impact on BC and contribute to FM growth (73, 74). Figure 3 shows different BC characteristics of preterm at birth, preterm at TEA, and full-term neonates.

### Altered body composition in the preschool age in preterm infants

The term “preschoolers” refers to the range of age from 3 to 5 years when children reach milestones in playing, learning, speaking, behavior, and moving (75). Among the few available studies evaluating BC on preschoolers born preterm, results are controversial.

Giannì et al. showed that very preterm neonates (mean GA at birth 30 ± 2 weeks, mean birth weight 1,152 ± 282 g) at TEA were lighter and shorter than term newborns, and with higher FM% and lower FFM by ADP measurements. At 5 years, compared with term, both male and female preterm infants remained significantly lighter (17.4 ± 2.3 kg vs. 20.2 ± 2.2 kg and 17.9 ± 3.1 kg vs. 19.9 ± 4.2 kg, respectively) and shorter (107 ± 5.1 cm vs. 113 ± 4.3 kg and 107.4 ± 4.4 kg vs. 111.6 ± 5.1 kg, respectively); males presented higher FM% (20.4 ± 5.0 vs. 17.9 ± 5.5) and lower FFM (13.9 ± 2.2 kg vs. 16.3 ± 1.8 kg, *p* < 0.001) compared to full term (76).

Diastolic blood pressure results higher in children born preterm (76). Pfister and colleagues highlighted that FM gain between term and 4 months corrected GA is associated with higher systolic and diastolic blood pressure at 4 years of age (77). Therefore, as suggested by the authors, longitudinal follow-up of growth’s quality may be predictive of later cardiometabolic outcomes.

Conversely, Scheurer et al. found that the differences in BC assessed by ADP at TEA between preterm (mean GA at birth 32 weeks, mean birth weight 1,843 g) and full-term children resolve in preschool age, with infancy FFM gains associated with preschool lean mass and prematurity not associated with FM evaluated at 4 years of age in both sexes (78). It should be noted that this study included only premature infants appropriate for GA.

Mihatsch et al. demonstrated that VLBW infants (mean GA at birth 29 ± 3 weeks) at 3 years have less FM% (19 ± 4 vs. 22 ± 4) and FFM in grams than full-term infants (9,796 ± 1,213 vs. 11,517 ± 1,234), without evidence of increased truncal fat in preterm infants evaluated by DXA (79).

Forsum et al. reported that, at 4 years, preterm children (GA at birth 23.3–36.9 weeks) are lighter and shorter and both early preterm girls and boys express less FM% than full-term infants by ADP measurement (25.3 ± 6.5 and 23.7 ± 3.5 vs. 25.1 ± 4.1 and 27.7 ± 4.5, respectively). Similarly, less FFM in kilograms both in early preterm girls and boys compared to the full-term was noticed (11.9 ± 1.4 and 13.4 ± 1.4 vs. 12.9 ± 1.6 and 14.0 ± 1.9, respectively). Moreover, GA was correlated with anthropometry and BC only in premature girls (80).

### The impact of gestational age at birth on body composition

The growth pattern is affected by GA. Ibáñez et al. showed that SGA infants have a peculiar catch-up growth compared with appropriate GA infants, characterized by shorter size and higher central adiposity according to DXA values at 2–4 years (81). The growth rate in FFM and FM% of SGA infants is higher than in Large for Gestational Age infants, defined as birth weight greater than the 90th percentile for age (82). Wells et al. speculated that poor fetal growth leads to an altered epigenetic programming of infants affecting both lean body mass and metabolic capacity with a consequent rapid catch-up growth, especially in terms of adiposity (27). Furthermore, during the early months of life feeding patterns may return the infant toward its genetic growth trajectory (83).

Fetal, rather than postnatal, FFM tissue accretion was associated with developmental progression (58). Moreover, prolonged invasive mechanical ventilation has been associated with skeletal muscle impairment detected with MRI (84). Lack of muscle mass is linked to insulin resistance due to reduced whole-body glucose uptake as muscle is an insulin target tissue (85). Conversely, higher FFM is associated with higher cognitive scores (21). Among ELBW preterm infants, LBW is strongly correlated with reduced lean mass in childhood from BC analysis by DXA (86). Postnatal FFM gain measured with ADP was positively associated with neuro-psychomotor development until preschool age, both in preterm (mean GA at birth 32 weeks, mean birth weight 1,843 g) infants (87) and very preterm (mean GA at birth 28.2 ± 2.2 weeks, mean birth weight 1,094 ± 257 g) (77).

### Sex differences in body composition of preterm newborn infants

Already in the fetal period, natural selection promotes the development of BC in a different way between the genders. According to specific evolutionary strategies, the growth is associated more strongly with lean mass development in males (to facilitate intermale competition and resource acquisition) and with fat mass development in females (to support elevated energy requirements during pregnancy and lactation) (27). In fact, female full-term born infants have a higher FM% than male full-term born infants (11.1 ± 4.1 vs. 9.6 ± 4.0, *p* < 0.001), while the males have higher mean FFM as well as higher birth weight (72).

Males and females respond differently to the intrauterine environment. The placenta’s ability to transport nutrients from mother to fetus, reflected in its size, allows optimal fetal growth. Male fetal growth is more rapid and invests in brain growth rather than placental growth. Consequently, there is a greater risk of undernutrition that may lead to LBW and, in the long-term, vulnerability to cardiovascular disorders (88).

Interestingly, Simon et al. found that male preterm infants (mean GA at birth 31.7 ± 2.4 weeks, mean birth weight 1,645 ± 501 g) have a significantly higher percentage of body fat than those full term (13.4 ± 4.2 vs. 10.1 ± 3.7, *p* < 0.001) at 6 weeks of postnatal age, using ADP to measure BC. Authors have hypothesized this is due to a lesser accretion in lean body mass in the first few weeks of extrauterine life (89). Dickson et al. observed that very preterm girls (born at < 30 weeks GA, birth weight < 1,500 g) have higher insulin secretion than boys born preterm and thus they are able to adapt more rapidly to the postnatal environment (90). Furthermore, preterm male infants also present a greater respiratory morbidity risk as surfactant production in fetal lungs appears later in male than in female, leading to lower airflow and increased resistance in the respiratory system (91). Therefore, preterm males are more severely ill than females during hospitalization and they are exposed to higher levels of stress-induced cortisol secretion, which may elicit higher rates of protein breakdown (89).

It was observed that maternal overweight or obesity status before pregnancy is associated with a higher risk of obesity in men at 1 year of age (92). Gestational diabetes also appears a risk factor for childhood overweight in boys but not in girls at 5–7 years (93).

## Early nutrition as programming for body composition

Early life nutrition represents an environmental stimulus applied at extrauterine life, which is critical for preterm infants. Energy and nutrients supplied may have a role in programming as regulating both quantity and quality of growth, metabolism, and immunity, with potential long-term effects (7, 94). Preterm neonates have rapid and exponential growth *ex utero*. Feeding them is challenging because nutritional needs are higher than those of full-term infants. Moreover, neonatal morbidity, increased respiratory work, and immaturity of the gastrointestinal tract may affect the achievement of adequate growth with an increased risk of postnatal growth failure (72).

The rate of weight gain in preterm infants is influenced by calories given, whereas gains in length and head circumference are influenced by the amount of protein (28). While head circumference has been positively correlated with improved cognitive outcomes (95), preterm infants developing faster weight gain experienced an increased risk of overweight and obesity, greater BF%, and higher blood pressure values (96).

According to ESPGHAN Committee on Nutrition, the preferred nutrition for premature infants is human milk (HM) (97). Expressed HM is the first choice for feeding preterm infants due to its beneficial effects on cardiovascular, neurological, and growth outcomes. When not available, the second choice is pasteurized donor HM (98). HM may be fortified with HM fortifiers to meet the high protein requirement of preterm infants in order to achieve optimal growth rates. In addition, the HM protein content of mothers who deliver prematurely declines over lactation, whereas fat and carbohydrate concentrations remain constant over time (99). When own mother milk is insufficient or non-available, if possible, pasteurized donor human milk should be chosen (100). If human milk cannot be chosen, the preterm formula should be used.

BC of preterm infants is influenced by nutritional intervention type (54). In a recent review, Giannì et al. showed that HM enhances protein use rather than their oxidation compared with formula in preterm neonates (101). Although HM is related to a slower weight gain compared to formula (102), it promotes anabolism and growth of quality tissues when nutritional needs are met (101). In a cohort of VLBW preterm infants fed with HM and assessed by ADP at TEA, Perrone et al. found lower protein oxidation for energy purposes, greater fat-free mass (2.05 ± 0.26 kg vs. 1.82 ± 0.35 kg, *p* < 0.01) and lower FM% (11.9 ± 3.9 vs. 15.1 ± 2.9, *p* < 0.01) compared with those fed with formula (103). Mol et al. demonstrated that VLBW preterm infants fed with HM shared similar body composition (15.7 ± 1.6% and 2.8 ± 0.3 kg, respectively) with full-term infants (14.5 ± 1.3%, *p* = 0.13 and 2.9 ± 0.3 kg, *p* = 0.88, respectively) at TEA, while VLBW infants fed with a formula developed higher fat mass evaluated by bioelectrical impedance analysis as compared with full term (16.5 ± 1.5% vs. 14.5 ± 1.3%, *p* < 0.01) (104).

The growth in preterm infants fed with HM is characterized by a preferential FFM deposition which, unlike fat gain, seems to contribute to the improvement of neurodevelopment outcomes (66, 87).

As previously discussed, the disadvantage in male preterm is multifactorial and boys require higher nutritional intake compared to girls to support optimal linear growth and BC (105). There is evidence that the composition of HM may differ by offspring sex. A recent narrative review stated that HM supplies more lipid and energy to boys and levels of hormones such as IGF and glucocorticoids are also reported to vary with sex. It seems, moreover, that the difference in nutritional intake by sex may already be influenced during pregnancy since men and women have different immunological profiles that could affect neonatal growth and health. There is evidence, for instance, that fetal sex influences maternal plasma cytokines and nitrates (NOx) in normal early gestation. In particular, women with a male fetus have higher IL1β (Interleukin 1 beta) and a lower IL13 (Interleukin 13) plasma levels, a lower capacity to counteract inflammation, and a higher plasma NOx with likely better dilator capacity. Data from normal pregnancies demonstrated that plasma testosterone concentrations are higher in males, being important not only for sex determination but also for sex differences in fetal growth. In addition, the male placenta from preterm delivery exhibits a higher level of inflammation, possibly because of a more robust maternal immune response to a male fetus (106, 107). Therefore, early nutrition could affect boys and girls differently, and further investigations are required in this field (108).

## Associations between preterm gut microbiota and body composition

Gut microbiota refer to bacteria, archaea, eukarya, and viruses living in the human intestinal tract. The composition of gut microbiota has been implicated in metabolic-immune homeostasis with potential effects on the growth and development of metabolic diseases (109, 110). The colonization of the intestinal tract starts during pregnancy and continues after birth both vertically (from the mother) and horizontally (from the environment) (111). The gut microbiota of a term vaginally delivered and exclusively breastfed infant is considered the gold standard for a healthy infant microbiota (112). Breast milk contributes to the maturation of the intestinal tract and the gut microbiota, and consequently to the immune system (113). The microbiota of breastfed infants mainly consists of *Bifidobacterium* and *Lactobacillus*. In contrast, the microbiome of formula-fed infants presents a relative abundance of Bacteroidetes and Firmicutes with increased *Clostridium difficile* (114). Human milk oligosaccharides (HMO) are fundamental bioactive components of breast milk. Their fermentation to short-chain fatty acids may modulate adiposity and satiety hormones with potential consequences on homeostasis. *Bifidobacterium spp*., abundant in vaginally born breastfed infants, are major HMO utilizers. Effects of *Bifidobacterium spp.* include the amelioration of diet-induced endotoxemia and inflammation and the deconjugation of bile acids influential to the host’s lipid metabolism and energy expenditure (115). In obese children, a decreased proportion of *Bifidobacterium spp.* has been associated with cesarean section delivery, not being breastfed, and antibiotic use (116). Indeed, the use of antibiotics with broad-spectrum has been associated with a delay in normal intestinal colonization (117).

In addition to delivery mode, early nutrition, and antibiotics treatment during neonatal care, GA is another factor driving gut microbiota development (116, 118).

Little is still known about the role of gut microbiota in preterm infants. However, in comparison to term infants, the gut microbiota of preterm infants is characterized by delayed colonization (low abundance) and limited microbial diversity (113). In their review, Westerbeek et al. concluded that colonization of *Bifidobacterium* and *Lactobacillus* is delayed in preterm infants, whereas colonization with potentially pathogenic bacteria is increased (117). The microbiota of hospitalized VLBW infants appeared mainly composed of Enterobacteriaceae (*Klebsiella* and *Escherichia* in particular) and Enterococcaceae (119, 120), differently from healthy term infants who are commonly characterized in early life by *Bifidobacterium*, Bacteroides, and *Clostridium* (121).

As PB alters gut microbiota development, an association among gut microbiota composition, nutrition, and infant growth has been suggested. The levels of Enterobacteriaceae and *Streptococcus* at 2 days of age and Bacteroides group at 10 days of age were found to affect weight gain at 1 month of age (112). Grier et al. distinguished three phases of microbiota development according to the relative dominant composition (Bacilli, Gammaproteobacteria, and Clostridia) strongly related to GA (29 weeks or less, 28–36 weeks, 33 weeks or later, respectively). *Staphylococcus*, *Clostridium*, and Enterococcus have been associated with an increased ratio between carbohydrates and total caloric intake, reflecting a potential change in the energy balance and an increased growth due to a greater expression of genes involved in lipid and carbohydrate metabolism (118).

Nevertheless, Stewart et al. showed that at 1–3 years, the preterm gut microbiome has a complexity comparable to healthy term infants regardless of antibiotic use and the presence of diseases. They supposed that introducing semisolid foods can promote the development and diversification of gut microbiota into the adult form (120). The gut microbiota of a term infant changes during the first year of life and becomes similar to adults between 1 and 4 years. Therefore, this period could represent a window of opportunity to modulate the gut microbiota to positively influence long-term health (122).

## Conclusion

PB disrupts the metabolic and endocrine maternal–placental unit. Preterm newborn infants are characterized by low body weight and lower fat mass than full-term born because of the interrupted supply of nutrients during intrauterine fetal growth. The postnatal adaptation to extrauterine life leads to the accretion of fat mass, aiming to improve thermoregulation and increase body energy stores. Therefore, at term equivalent age, body fat percentage is more represented in preterm as compared with full-term born neonates, causing a predisposition to developing metabolic syndrome and cardiovascular diseases in childhood and adulthood.

Both quantity and quality of growth are regulated by several factors such as fetal programming, early nutrition, and gut microbiota. Moreover, the differences in body composition have been reported between males and females born preterm in a not consistent way across the studies; the mechanisms are likely to be multifactorial including the differential activity of the placenta and endocrine activation in response to stress. As gain in weight alone is not an optimal indicator of nutritional status, analysis of body composition represents a potentially useful tool to predict later metabolic and cardiovascular risk allowing detection of the quality of growth by differentiating between fat and lean mass through longitudinal measurements.

Body composition analysis provides the missing key to optimally manage preterm neonates. Indeed, the study of body compartments may reveal a “dys-phenotype” that predisposes to later metabolic risk, even during the first phases of life.

## Author contributions

AC: conceptualization and writing the original draft. AC and AS: methodology. AC, AS, MP, GV, FS, and EM: data curation. FS, RC, and MS: supervision. All authors have supervised and contributed to the writing, review, and editing of the final version of this manuscript.

## Abbreviations

2-C: two-compartment model
3-C: three-compartment model
4-C: four-compartment model
ADP: air-displacement plethysmography
BC: body composition
BMI: body mass index
BW: birth weight
DOHaD: Developmental Origins of Health and Disease
DXA: dual-energy x-ray absorptiometry
ELBW: extremely low birth weight
ESPGHAN: European Society for Pediatric Gastroenterology Hepatology and Nutrition
EUGR: extrauterine growth restriction
FAs: fatty acids
FFM: fat-free mass
FM%: fat mass percentage
FM: fat mass
FP: fetal programming
GA: gestational age
HM: human milk
IUGR: intrauterine growth restriction
LBM: lean body mass
LBW: low birth weight
MRI: magnetic resonance imaging
NOx: nitrates
PB: preterm birth
SGA: small for gestational age
TEA: term equivalent age
US: ultrasound imaging
VLBW: very low birth weight.

## References

[1] ICD-10 Version:2016. *Apps.Who.Int.* (2022). Available online at: http://apps.who.int/classifications/icd10/browse/2016/en#!/P05.9 (accessed March 16, 2022)

[2] Yumani D Lafeber H van Weissenbruch M. IGF-I, growth, and body composition in preterm infants up to term equivalent age. *J Endoc Soc.* (2021) 5:7. 10.1210/jendso/bvab089 34159288 PMC8212689

[3] Gallagher D Andres A Fields D Evans W Kuczmarski R Lowe W Body composition measurements from birth through 5 years: challenges, gaps, and existing & emerging technologies—a national institutes of health workshop. *Obesity Rev.* (2020) 21:8. 10.1111/obr.13033 32314544 PMC7875319

[4] Sacchi C Marino C Nosarti C Vieno A Visentin S Simonelli A. Association of intrauterine growth restriction and small for gestational age status with childhood cognitive outcomes. *JAMA Pediatr.* (2020) 174:772. 10.1001/jamapediatrics.2020.1097 32453414 PMC7251506

[5] Fenton T Cormack B Goldberg D Nasser R Alshaikh B Eliasziw M “Extrauterine growth restriction” and “postnatal growth failure” are misnomers for preterm infants. *J Perinatol.* (2020) 40:704–14. 10.1038/s41372-020-0658-5 32214217

[6] Thomas E Al Saud N Durighel G Frost G Bell J. The effect of preterm birth on adiposity and metabolic pathways and the implications for later life. *Clin Lipidology.* (2012) 7:275–88. 10.2217/clp.12.32

[7] Lucas A Fewtrell M Cole T. Fetal origins of adult disease—the hypothesis revisited. *BMJ.* (1999) 319:245–9. 10.1136/bmj.319.7204.245 10417093 PMC1116334

[8] Barker D. The fetal and infant origins of adult disease. *BMJ.* (1990) 301:1111. 10.1136/bmj.301.6761.1111 2252919 PMC1664286

[9] Fall C. Developmental origins of cardiovascular disease, type 2 diabetes and obesity in humans. *Early Life Origins Health Dis.* (2013) 18:185–97.

[10] Abitbol C Rodriguez M. The long-term renal and cardiovascular consequences of prematurity. *Nat Rev Nephrol.* (2012) 8:265–74. 10.1038/nrneph.2012.38 22371245

[11] Kistner A Celsi G Vanpée M Jacobson S. Increased systolic daily ambulatory blood pressure in adult women born preterm. *Pediatr Nephrol.* (2004) 20:232–3. 10.1007/s00467-004-1717-4 15583945

[12] Ligi I Grandvuillemin I Andres V Dignat-George F Simeoni U. Low birth weight infants and the developmental programming of hypertension: a focus on vascular factors. *Semin Perinatol.* (2010) 34:188–92. 10.1053/j.semperi.2010.02.002 20494734

[13] Gutiérrez-Arzapalo P Rodríguez-Rodríguez P Ramiro-Cortijo D López de Pablo Á López-Giménez M Condezo-Hoyos L Role of fetal nutrient restriction and postnatal catch-up growth on structural and mechanical alterations of rat aorta. *J Physiol.* (2018) 596:5791–806. 10.1113/JP275030 29277911 PMC6265550

[14] Norman M. Low birth weight and the developing vascular tree: a systematic review. *Acta Paediatr.* (2008) 97(9):1165–72. 10.1111/j.1651-2227.2008.00904.x 18554273

[15] Brenner B Chertow G. Congenital oligonephropathy and the etiology of adult hypertension and progressive renal injury. *Am J Kidney Dis.* (1994) 23:171–5. 10.1016/S0272-6386(12)80967-X 8311070

[16] Li E. Chromatin modification and epigenetic reprogramming in mammalian development. *Nat Rev Genet.* (2002) 3:662–73. 10.1038/nrg887 12209141

[17] Rees W Hay S Brown D Antipatis C Palmer R. Maternal protein deficiency causes hypermethylation of DNA in the livers of rat fetuses. *J Nutr.* (2000) 130:1821–6. 10.1093/jn/130.7.1821 10867057

[18] DeFreitas M Katsoufis C Benny M Young K Kulandavelu S Ahn H Educational review: the impact of perinatal oxidative stress on the developing kidney. *Front Pediatr.* (2022) 2022:10. 10.3389/fped.2022.853722 35844742 PMC9279889

[19] Pennathur S Heinecke J. Oxidative stress and endothelial dysfunction in vascular disease. *Curr Diab Rep.* (2007) 7:257–64. 10.1007/s11892-007-0041-3 17686400

[20] Rodríguez-Rodríguez P Ramiro-Cortijo D Reyes-Hernández C López de Pablo A González M Arribas S. Implication of oxidative stress in fetal programming of cardiovascular disease. *Front Physiol.* (2018) 9:602. 10.3389/fphys.2018.00602 29875698 PMC5974054

[21] Plummer E Wang Q Larson-Nath C Scheurer J Ramel S. Body composition and cognition in preschool-age children with congenital gastrointestinal anomalies. *Early Hum Dev.* (2019) 129:5–10. 10.1016/j.earlhumdev.2018.12.001 30562643 PMC6382521

[22] Cooke R Griffin I McCormick K. Adiposity is not altered in preterm infants fed with a nutrient-enriched formula after hospital discharge. *Pediatr Res.* (2010) 67:660–4. 10.1203/PDR.0b013e3181da8d01 20216105

[23] Ong K Kennedy K Castañeda-Gutiérrez E Forsyth S Godfrey K Koletzko B Postnatal growth in preterm infants and later health outcomes: a systematic review. *Acta Paediatr.* (2015) 104:974–86. 10.1111/apa.13128 26179961 PMC5054880

[24] Victora C Barros F. Commentary: the catch-up dilemma— relevance of leitch’s ‘low–high’ pig to child growth in developing countries. *Int J Epidemiol.* (2001) 30:217–20. 10.1093/ije/30.2.217 11369717

[25] de Wit C Sas T Wit J Cutfield W. Patterns of catch-up growth. *J Pediatr.* (2013) 162:415–20. 10.1016/j.jpeds.2012.10.014 23153864

[26] Andersen L Ängquist L Eriksson J Forsen T Gamborg M Osmond C Birth weight, childhood body mass index and risk of coronary heart disease in adults: combined historical cohort studies. *PLoS One.* (2010) 5:e14126. 10.1371/journal.pone.0014126 21124730 PMC2993956

[27] Wells J Chomtho S Fewtrell M. Programming of body composition by early growth and nutrition. *Proc Nutr Soc.* (2007) 66:423–34. 10.1017/S0029665107005691 17637095

[28] Sauer P. Can extrauterine growth approximate intrauterine growth? Should it? *Am J Clin Nutr.* (2007) 85:608S–13S. 10.1093/ajcn/85.2.608S 17284764

[29] Fall C Sachdev H Osmond C Lakshmy R Biswas S Prabhakaran D Adult metabolic syndrome and impaired glucose tolerance are associated with different patterns of BMI gain during infancy. *Diab Care.* (2008) 31:2349–56. 10.2337/dc08-0911 18835958 PMC2584194

[30] Adair L Fall C Osmond C Stein A Martorell R Ramirez-Zea M Associations of linear growth and relative weight gain during early life with adult health and human capital in countries of low and middle income: findings from five birth cohort studies. *Lancet.* (2013) 382:525–34. 10.1016/S0140-6736(13)60103-8 23541370 PMC3744751

[31] Okada T Takahashi S Nagano N Yoshikawa K Usukura Y Hosono S. Early postnatal alteration of body composition in preterm and small-for-gestational-age infants: implications of catch-up fat. *Pediatr Res.* (2014) 77:136–42. 10.1038/pr.2014.164 25310764

[32] Rice M Valentine C. Neonatal body composition. *Nutr Clin Pract.* (2015) 30:625–32. 10.1177/0884533615578917 25908606

[33] Parlapani E Agakidis C Karagiozoglou–Lampoudi T. Anthropometry and body composition of preterm neonates in the light of metabolic programming. *J Am College Nutr.* (2018) 37:350–9. 10.1080/07315724.2017.1400479 29425475

[34] Patel AL Engstrom JL Meier PP Kimura RE. Accuracy of methods for calculating postnatal growth velocity for extremely low birth weight infants. *Pediatrics.* (2005) 116:1466–73. 10.1542/peds.2004-1699 16322172

[35] Fenton TR Chan HT Madhu A Griffin IJ Hoyos A Ziegler EE Preterm infant growth velocity calculations: a systematic review. *Pediatrics.* (2017) 139:3. 10.1542/peds.2016-2045 28246339

[36] Griffin IJ Tancredi DJ Bertino E Lee HC Profit J. Postnatal growth failure in very low birthweight infants born between 2005 and 2012. *Arch Dis Child Fetal Neonatal Ed.* (2016) 101:F50–5. 10.1136/archdischild-2014-308095 26201534

[37] González Jiménez E. Body composition: assessment and clinical value. *Endocrinología y Nutr.* (2013) 60:69–75. 10.1016/j.endoen.2012.04.015 22704270

[38] Kiger J Taylor S Wagner C Finch C Katikaneni L. Preterm infant body composition cannot be accurately determined by weight and length. *J Neonat Perinat Med.* (2016) 9:285–90. 10.3233/NPM-16915125 27589548

[39] Ramel S Zhang L Misra S Anderson C Demerath E. Do anthropometric measures accurately reflect body composition in preterm infants? *Pediatr Obesity.* (2016) 12:72–7. 10.1111/ijpo.12181 27635625

[40] Marra M Sammarco R De Lorenzo A Iellamo F Siervo M Pietrobelli A Assessment of body composition in health and disease using bioelectrical impedance analysis (BIA) and dual energy X-ray absorptiometry (DXA): a critical overview. *Contrast Med Mol Imag.* (2019) 2019:1–9. 10.1155/2019/3548284 31275083 PMC6560329

[41] Wang Z Pierson R Heymsfield S. The five-level model: a new approach to organizing body-composition research. *Am J Clin Nutr.* (1992) 56:19–28. 10.1093/ajcn/56.1.19 1609756

[42] Heymsfield S Wang Z Baumgartner R Ross R. Human body composition: advances in models and methods. *Ann Rev Nutr.* (1997) 17:527–58. 10.1146/annurev.nutr.17.1.527 9240939

[43] Ellis K Yao M Shypailo R Urlando A Wong W Heird W. Body-composition assessment in infancy: air-displacement plethysmography compared with a reference 4-compartment model. *Am J Clin Nutr.* (2007) 85:90–5. 10.1093/ajcn/85.1.90 17209182

[44] Andrews E Beattie R Johnson M. Measuring body composition in the preterm infant: evidence base and practicalities. *Clin Nutr.* (2019) 38:2521–30. 10.1016/j.clnu.2018.12.033 30737045

[45] Pourhassan M Schautz B Braun W Gluer C Bosy-Westphal A Müller M. Impact of body-composition methodology on the composition of weight loss and weight gain. *Eur J Clin Nutr.* (2013) 67:446–54. 10.1038/ejcn.2013.35 23422922

[46] Roggero P Giannì M Amato O Piemontese P Morniroli D Wong W Evaluation of air-displacement plethysmography for body composition assessment in preterm infants. *Pediatr Res.* (2012) 72:316–20. 10.1038/pr.2012.75 22669294

[47] Roggero P Giannì M Amato O Agosti M Fumagalli M Mosca F. Measuring the body composition of preterm and term neonates: from research to clinical applications. *J Pediatr Gastroenterol Nutr.* (2007) 45:S159–62. 10.1097/01.mpg.0000302964.85922.1a 18185084

[48] Ma G Yao M Liu Y Lin A Zou H Urlando A Validation of a new pediatric air-displacement plethysmograph for assessing body composition in infants. *Am J Clin Nutr.* (2004) 79:653–60. 10.1093/ajcn/79.4.653 15051611

[49] Demerath E Johnson W Davern B Anderson C Shenberger J Misra S New body composition reference charts for preterm infants. *Am J Clin Nutr.* (2016) 105:70–7. 10.3945/ajcn.116.138248 27806978

[50] Mazahery H von Hurst PR McKinlay CJ Cormack BE Conlon CA. Air displacement plethysmography (pea pod) in full-term and pre-term infants: a comprehensive review of accuracy, reproducibility, and practical challenges. *Mate Health Neonatol Perinatol.* (2018) 4:12. 10.1186/s40748-018-0079-z 29951209 PMC6011189

[51] Shen W Wang Z Punyanita M Lei J Sinav A Kral J Adipose tissue quantification by imaging methods: a proposed classification. *Obesity Res.* (2003) 11:5–16. 10.1038/oby.2003.3 12529479 PMC1894646

[52] Johnson M Wootton S Leaf A Jackson A. Preterm birth and body composition at term equivalent age: a systematic review and meta-analysis. *Pediatrics.* (2012) 130:e640–9. 10.1542/peds.2011-3379 22891222

[53] Valentine R Misic M Kessinger R Mojtahedi M Evans E. Location of body fat and body size impacts DXA soft tissue measures: a simulation study. *Eur J Clin Nutr.* (2007) 62:553–9. 10.1038/sj.ejcn.1602770 17457339

[54] Strydom K Van Niekerk E Dhansay M. Factors affecting body composition in preterm infants: assessment techniques and nutritional interventions. *Pediatr Neonatol.* (2019) 60:121–8. 10.1016/j.pedneo.2017.10.007 29239827

[55] Scholten RR Pillen S Verrips A Zwarts MJ. Quantitative ultrasonography of skeletal muscles in children: normal values. *Muscle Nerve.* (2003) 27:693–8. 10.1002/mus.10384 12766980

[56] Bertini G Elia S Dani C. Using ultrasound to examine muscle mass in preterm infants at term-equivalent age. *Eur J Pediatr.* (2020) 180:461–8. 10.1007/s00431-020-03846-7 33083899

[57] Kuzawa C. Adipose tissue in human infancy and childhood: an evolutionary perspective. *Am J Phys Anthropol.* (1998) 107:177–209. 10.1002/(SICI)1096-8644(1998)107:27+<177::AID-AJPA7>3.0.CO;2-B 9881526

[58] Abera M Tesfaye M Admassu B Hanlon C Ritz C Wibaek R Body composition during early infancy and developmental progression from 1 to 5 years of age: the infant anthropometry and body composition (iABC) cohort study among ethiopian children. *Br J Nutr.* (2018) 119:1263–73. 10.1017/S000711451800082X 29770755

[59] Herrera E Desoye G. Maternal and fetal lipid metabolism under normal and gestational diabetic conditions. *Hormone Mol Biol Clin Invest.* (2016) 26:2. 10.1515/hmbci-2015-0025 26351960

[60] Desoye G Herrera E. Adipose tissue development and lipid metabolism in the human fetus: the 2020 perspective focusing on maternal diabetes and obesity. *Prog Lipid Res.* (2021) 81:101082. 10.1016/j.plipres.2020.101082 33383022

[61] Ziegler EE O’Donnell AM Nelson SE Fomon SJ. Body composition of the reference fetus. *Growth.* (1976) 40:329–41. 1010389

[62] Neal-Kluever A Fisher J Grylack L Kakiuchi-Kiyota S Halpern W. Physiology of the neonatal gastrointestinal system relevant to the disposition of orally administered medications. *Drug Metab Disposit.* (2018) 47:296–313. 10.1124/dmd.118.084418 30567878

[63] Sarr O Yang K Regnault T. In uteroprogramming of later adiposity: the role of fetal growth restriction. *J Preg.* (2012) 2012:1–10. 10.1155/2012/134758 23251802 PMC3518064

[64] Bortolotto C Santos I dos Santos Vaz J Matijasevich A Barros A Barros F Prematurity and body composition at 6, 18, and 30 years of age: pelotas (Brazil) 2004, 1993, and 1982 birth cohorts. *BMC Public Health.* (2021) 21:321. 10.1186/s12889-021-10368-w 33563247 PMC7871570

[65] Giannì M Roggero P Liotto N Taroni F Polimeni A Morlacchi L Body composition in late preterm infants according to percentile at birth. *Pediatr Res.* (2015) 79:710–5. 10.1038/pr.2015.273 26717003

[66] Roggero P Giannì M Amato O Orsi A Piemontese P Morlacchi L Is term newborn body composition being achieved postnatally in preterm infants? *Early Hum Dev.* (2009) 85:349–52. 10.1016/j.earlhumdev.2008.12.011 19162413

[67] Chmielewska A Farooqi A Domellöf M Ohlund I. Lean tissue deficit in preterm infants persists up to 4 months of age: results from a swedish longitudinal study. *Neonatology.* (2019) 117:80–7. 10.1159/000503292 31822002

[68] Bruckner M Khan Z Binder C Morris N Windisch B Holasek S Extremely preterm infants have a higher fat mass percentage in comparison to very preterm infants at term-equivalent age. *Front Pediatr.* (2020) 8:61. 10.3389/fped.2020.00061 32219084 PMC7078645

[69] Uthaya S Thomas E Hamilton G Doré C Bell J Modi N. Altered Adiposity after extremely preterm birth. *Pediatr Res.* (2005) 57:211–5. 10.1203/01.PDR.0000148284.58934.1C 15611357

[70] Giannì M Mora S Roggero P Amato O Piemontese P Orsi A Regional fat distribution in children born preterm evaluated at school age. *J Pediatr Gastroenterol Nutr.* (2008) 46:232–5. 10.1097/MPG.0b013e31814d4df9 18223391

[71] Ramel S Gray H Ode K Younge N Georgieff M Demerath E. Body composition changes in preterm infants following hospital discharge. *J Pediatr Gastroenterol Nutr.* (2011) 53:333–8. 10.1097/MPG.0b013e3182243aa7 21602717 PMC7680641

[72] Wiechers C Bernhard W Goelz R Poets C Franz A. Optimizing early neonatal nutrition and dietary pattern in premature infants. *Int J Environ Res Public Health.* (2021) 18:7544. 10.3390/ijerph18147544 34300000 PMC8304391

[73] Orsso C Colin-Ramirez E Field C Madsen K Prado C Haqq A. Adipose tissue development and expansion from the womb to adolescence: an overview. *Nutrients.* (2020) 12:2735. 10.3390/nu12092735 32911676 PMC7551046

[74] Hernandez T Van Pelt R Anderson M Reece M Reynolds R de la Houssaye B Women with gestational diabetes mellitus randomized to a higher–complex carbohydrate/low-fat diet manifest lower adipose tissue insulin resistance, inflammation, glucose, and free fatty acids: a pilot study. *Diab Care.* (2015) 39:39–42. 10.2337/dc15-0515 26223240 PMC4686845

[75] CDC. *Preschoolers (3-5 years of age).* (2022). Available online at: https://www.cdc.gov/ncbddd/childdevelopment/positiveparenting/preschoolers.html (accessed March 16, 2022)

[76] Giannì M Roggero P Piemontese P Morlacchi L Bracco B Taroni F Boys who are born preterm show a relative lack of fat-free mass at 5 years of age compared to their peers. *Acta Paediatr.* (2014) 104:e119–23. 10.1111/apa.12856 25382273

[77] Pfister K Zhang L Miller N Ingolfsland E Demerath E Ramel S. Early body composition changes are associated with neurodevelopmental and metabolic outcomes at 4 years of age in very preterm infants. *Pediatr Res.* (2018) 84:713–8. 10.1038/s41390-018-0158-x 30188501 PMC6294700

[78] Scheurer J Zhang L Gray H Weir K Demerath E Ramel S. Body composition trajectories from infancy to preschool in children born premature versus full-term. *J Pediatr Gastroenterol Nutr.* (2017) 64:e147–53. 10.1097/MPG.0000000000001494 28045768

[79] Mihatsch W Dorronsoro Martín I Barrios-Sabador V Couce M Martos-Moreno G Argente J Bone mineral density, body composition, and metabolic health of very low birth weight infants fed in hospital following current macronutrient recommendations during the first 3 years of life. *Nutrients.* (2021) 13:1005. 10.3390/nu13031005 33804764 PMC8003951

[80] Forsum E Flinke E Olhager E. Premature birth was not associated with increased body fatness in four-year-old boys and girls. *Acta Paediatr.* (2019) 109:327–31. 10.1111/apa.14990 31461786

[81] Ibáñez L Ong K Dunger D de Zegher F. Early development of adiposity and insulin resistance after catch-up weight gain in small-for-gestational-age children. *J Clin Endocrinol Metab.* (2006) 91:2153–8. 10.1210/jc.2005-2778 16537681

[82] Larsson A Ottosson P Törnqvist C Olhager E. Body composition and growth in full-term small for gestational age and large for gestational age Swedish infants assessed with air displacement plethysmography at birth and at 3-4 months of age. *PLoS One.* (2019) 14:e0207978. 10.1371/journal.pone.0207978 31091240 PMC6519902

[83] Ong K Dunger D. Birth weight, infant growth and insulin resistance. *Eur J Endocrinol.* (2004) 151:U131–9. 10.1530/eje.0.151u131 15554898

[84] Dassios T Kaltsogianni O Krokidis M Hickey A Greenough A. Deltoid muscle morphometry as an index of impaired skeletal muscularity in neonatal intensive care. *Eur J Pediatr.* (2018) 177:507–12. 10.1007/s00431-018-3090-5 29350333

[85] Burrows R Correa-Burrows P Reyes M Blanco E Albala C Gahagan S. Low muscle mass is associated with cardiometabolic risk regardless of nutritional status in adolescents: a cross-sectional study in a chilean birth cohort. *Pediatr Diab.* (2017) 18:895–902. 10.1111/pedi.12505 28145023 PMC5538898

[86] Stutte S Gohlke B Peiler A Schreiner F Born M Bartmann P Impact of early nutrition on body composition in children aged 9.5 years born with extremely low birth weight. *Nutrients.* (2017) 9:124. 10.3390/nu9020124 28208596 PMC5331555

[87] Scheurer J Zhang L Plummer E Hultgren S Demerath E Ramel S. Body composition changes from infancy to 4 years and associations with early childhood cognition in preterm and full-term children. *Neonatology.* (2018) 114:169–76. 10.1159/000487915 29898453 PMC6083858

[88] Eriksson J Kajantie E Osmond C Thornburg K Barker D. Boys live dangerously in the womb. *Am J Hum Biol.* (2009) 22:330–5. 10.1002/ajhb.20995 19844898 PMC3923652

[89] Simon L Borrego P Darmaun D Legrand A Rozé J Chauty-Frondas A. Effect of sex and gestational age on neonatal body composition. *Br J Nutr.* (2012) 109:1105–8. 10.1017/S0007114512002991 22784704

[90] Dickson J Chase J Pretty C Gunn C Alsweiler J. Hyperglycaemic preterm babies have sex differences in insulin secretion. *Neonatology.* (2015) 108:93–8. 10.1159/000381206 26068110

[91] Townsel C Emmer S Campbell W Hussain N. Gender differences in respiratory morbidity and mortality of preterm neonates. *Front Pediatr.* (2017) 5:6. 10.3389/fped.2017.00006 28194395 PMC5276811

[92] Bridgman S Azad M Persaud R Chari R Becker A Sears M Impact of maternal pre-pregnancy overweight on infant overweight at 1 year of age: associations and sex-specific differences. *Pediatr Obesity.* (2018) 13:579–89. 10.1111/ijpo.12291 29797797

[93] Le Moullec N Fianu A Maillard O Chazelle E Naty N Schneebeli C Sexual dimorphism in the association between gestational diabetes mellitus and overweight in offspring at 5-7 years: the OBEGEST cohort study. *PLoS One.* (2018) 13:e0195531. 10.1371/journal.pone.0195531 29621322 PMC5886576

[94] Hanson C Sundermeier J Dugick L Lyden E Anderson-Berry A. Implementation, process, and outcomes of nutrition best practices for infants <1500 g. *Nutr Clin Pract.* (2011) 26:614–24. 10.1177/0884533611418984 21947645

[95] Leppänen M Lapinleimu H Lind A Matomäki J Lehtonen L Haataja L Antenatal and postnatal growth and 5-year cognitive outcome in very preterm infants. *Pediatrics.* (2014) 133:63–70. 10.1542/peds.2013-1187 24344103

[96] Wang G Johnson S Gong Y Polk S Divall S Radovick S Weight gain in infancy and overweight or obesity in childhood across the gestational spectrum: a prospective birth cohort study. *Sci Rep.* (2016) 6:29867. 10.1038/srep29867 27417566 PMC4945912

[97] Lapillonne A Bronsky J Campoy C Embleton N Fewtrell M Fidler Mis N Feeding the late and moderately preterm infant: a position paper of the european society for paediatric gastroenterology, hepatology and nutrition committee on nutrition. *J Pediatr Gastroenterol Nutr.* (2019) 69:259–70. 10.1097/MPG.0000000000002397 31095091

[98] Kumar R Singhal A Vaidya U Banerjee S Anwar F Rao S. Optimizing nutrition in preterm low birth weight infants—consensus summary. *Front Nutr.* (2017) 4:20. 10.3389/fnut.2017.00020 28603716 PMC5445116

[99] Lönnerdal B Erdmann P Thakkar S Sauser J Destaillats F. Longitudinal evolution of true protein, amino acids and bioactive proteins in breast milk: a developmental perspective. *J Nutr Biochem.* (2017) 41:1–11. 10.1016/j.jnutbio.2016.06.001 27771491

[100] Parker MG Stellwagen LM Noble L Kim JH Poindexter BB Puopolo KM. Promoting human milk and breastfeeding for the very low birth weight infant. *Pediatrics.* (2021) 148:e2021054272. 10.1542/peds.2021-054272 34635582

[101] Gianni M Roggero P Mosca F. Human milk protein vs. formula protein and their use in preterm infants. *Curr Opin Clin Nutr Metab Care.* (2019) 22:76–81. 10.1097/MCO.0000000000000528 30407223

[102] Cerasani J Ceroni F De Cosmi V Mazzocchi A Morniroli D Roggero P Human milk feeding and preterm infants’ growth and body composition: a literature review. *Nutrients.* (2020) 12:1155. 10.3390/nu12041155 32326178 PMC7230190

[103] Perrone M Menis C Piemontese P Tabasso C Mallardi D Orsi A Energy expenditure, protein oxidation and body composition in a cohort of very low birth weight infants. *Nutrients.* (2021) 13:3962. 10.3390/nu13113962 34836218 PMC8620881

[104] Mól N Zasada M Kwinta P. Does type of feeding affect body composition in very low birth weight infants? – A prospective cohort study. *Pediatr Neonatol.* (2019) 60:135–40. 10.1016/j.pedneo.2018.04.010 29784603

[105] O’Driscoll D McGovern M Greene C Molloy E. Gender disparities in preterm neonatal outcomes. *Acta Paediatr.* (2018) 107:1494–9. 10.1111/apa.14390 29750838

[106] Ramiro-Cortijo D de la Calle M Böger R Hannemann J Lüneburg N López-Giménez M Male fetal sex is associated with low maternal plasma anti-inflammatory cytokine profile in the first trimester of healthy pregnancies. *Cytokine.* (2020) 136:155290. 10.1016/j.cyto.2020.155290 32956948

[107] Gila-Diaz A Arribas S Algara A Martín-Cabrejas M López de Pablo Á Sáenz de Pipaón M A review of bioactive factors in human breastmilk: a focus on prematurity. *Nutrients.* (2019) 11:1307. 10.3390/nu11061307 31185620 PMC6628333

[108] Tottman A Oliver C Alsweiler J Cormack B. Do preterm girls need different nutrition to preterm boys? Sex-specific nutrition for the preterm infant. *Pediatr Res.* (2020) 89:313–7. 10.1038/s41390-020-01252-1 33184497

[109] Jian C Carpén N Helve O de Vos W Korpela K Salonen A. Early-life gut microbiota and its connection to metabolic health in children: perspective on ecological drivers and need for quantitative approach. *EBioMedicine.* (2021) 69:103475. 10.1016/j.ebiom.2021.103475 34256346 PMC8324810

[110] Cardinelli C Sala P Alves C Torrinhas R Waitzberg D. Influence of intestinal microbiota on body weight gain: a narrative review of the literature. *Obesity Surg.* (2014) 25:346–53. 10.1007/s11695-014-1525-2 25511750

[111] Moore R Townsend S. Temporal development of the infant gut microbiome. *Open Biol.* (2019) 9:190128. 10.1098/rsob.190128 31506017 PMC6769289

[112] Arboleya S Martinez-Camblor P Solís G Suárez M Fernández N de los Reyes-Gavilán C Intestinal microbiota and weight-gain in preterm neonates. *Front Microbiol.* (2017) 8:183. 10.3389/fmicb.2017.00183 28228752 PMC5296308

[113] Henderickx J Zwittink R van Lingen R Knol J Belzer C. The preterm gut microbiota: an inconspicuous challenge in nutritional neonatal care. *Front Cell Infect Microbiol.* (2019) 9:85. 10.3389/fcimb.2019.00085 31001489 PMC6454191

[114] Granger C Embleton N Palmer J Lamb C Berrington J Stewart C. Maternal breastmilk, infant gut microbiome and the impact on preterm infant health. *Acta Paediatr.* (2020) 110:450–7. 10.1111/apa.15534 33245565

[115] Chambers E Preston T Frost G Morrison D. Role of gut microbiota-generated short-chain fatty acids in metabolic and cardiovascular health. *Curr Nutr Rep.* (2018) 7:198–206. 10.1007/s13668-018-0248-8 30264354 PMC6244749

[116] Korpela K de Vos W. Early life colonization of the human gut: microbes matter everywhere. *Curr Opin Microbiol.* (2018) 44:70–8. 10.1016/j.mib.2018.06.003 30086431

[117] Westerbeek E van den Berg A Lafeber H Knol J Fetter W van Elburg R. The intestinal bacterial colonisation in preterm infants: a review of the literature. *Clin Nutr.* (2006) 25:361–8. 10.1016/j.clnu.2006.03.002 16677741

[118] Grier A Qiu X Bandyopadhyay S Holden-Wiltse J Kessler H Gill A Impact of prematurity and nutrition on the developing gut microbiome and preterm infant growth. *Microbiome.* (2017) 5:158. 10.1186/s40168-017-0377-0 29228972 PMC5725645

[119] Patel A Mutlu E Sun Y Koenig L Green S Jakubowicz A Longitudinal survey of microbiota in hospitalized preterm very-low-birth-weight infants. *J Pediatr Gastroenterol Nutr.* (2016) 62:292–303. 10.1097/MPG.0000000000000913 26230901 PMC4724288

[120] Stewart C Embleton N Marrs E Smith D Nelson A Abdulkadir B Temporal bacterial and metabolic development of the preterm gut reveals specific signatures in health and disease. *Microbiome.* (2016) 4:67. 10.1186/s40168-016-0216-8 28034304 PMC5200962

[121] Thompson-Chagoyán O Maldonado J Gil A. Colonization and impact of disease and other factors on intestinal microbiota. *Digest Dis Sci.* (2007) 52:2069–77. 10.1007/s10620-006-9285-z 17420934

[122] Wopereis H Oozeer R Knipping K Belzer C Knol J. The first thousand days - intestinal microbiology of early life: establishing a symbiosis. *Pediatr Allergy Immunol.* (2014) 25:428–38. 10.1111/pai.12232 24899389

